# Leadership and governance of community health worker programmes at scale: a cross case analysis of provincial implementation in South Africa

**DOI:** 10.1186/s12939-017-0565-3

**Published:** 2017-09-15

**Authors:** Helen Schneider, Nonhlanhla Nxumalo

**Affiliations:** 1 School of Public Health & SAMRC/UWC Health Services to Systems Unit, University of the Western Cape, Robert Sobukwe Road, Bellville, Cape Town 7535 South Africa; 2 Centre for Health Policy/MRC Health Policy Research Group, School of Public Health, Faculty of Health Sciences, University of the Witwatersrand, Johannesburg, South Africa

**Keywords:** Community systems, Community health workers, Community health system strengthening, National CHW programmes, Governance, Leadership, Stewardship, Strategic management

## Abstract

**Background:**

National community health worker (CHW) programmes are returning to favour as an integral part of primary health care systems, often on the back of pre-existing community based initiatives. There are significant challenges to the integration and support of such programmes, and they require coordination and stewardship at all levels of the health system. This paper explores the leadership and governance tasks of large-scale CHW programmes at sub-national level, through the case of national reforms to South Africa’s community based sector, referred to as the Ward Based Outreach Team (WBOT) strategy.

**Methods:**

A cross case analysis of leadership and governance roles, drawing on three case studies of adoption and implementation of the WBOTs strategy at provincial level (Western Cape, North West and Gauteng) was conducted. The primary case studies mapped system components and assessed implementation processes and contexts. They involved teams of researchers and over 200 interviews with stakeholders from senior to frontline, document reviews and analyses of routine data. The secondary, cross case analysis specifically focused on the issues and challenges facing, and strategies adopted by provincial and district policy makers and managers, as they engaged with the new national mandate. From this key sub-national leadership and governance roles were formulated.

**Results:**

Four key roles are identified and discussed:

1. Negotiating a fit between national mandates and provincial and district histories and strategies of community based services
2. Defining new organisational and accountability relationships between CHWs, local health services, communities and NGOs
3. Revising and developing new aligned and integrated planning, human resource, financing and information systems
4. Leading change by building new collective visions, mobilising political, including budgetary, support and designing implementation strategies.

**Conclusions:**

This analysis, from real-life systems, adds to understanding of the processes involved in developing CHW programmes at scale, and specifically the negotiated and multilevel nature of leadership and governance in such programmes, spanning analytic, managerial, technical and political roles.

## Introduction

Community health workers (CHWs) have a long and varied history in health systems, recently regaining attention [1]. There is well-established evidence on the role of CHWs and community based health action in improved health outcomes, and increasing consensus on their importance in primary health care (PHC) systems and in achieving universal health coverage [2, 3]. CHW programmes promote equity by increasing access to health care in remote areas, and by playing a mediating role between the formal health system and marginalised populations [4]. A growing list of low and middle income countries, such as Brazil, Ethiopia, Malawi, Bangladesh, Nepal, amongst others, have recognised national CHW programmes [5], while others are formulating or revising national CHW policies [6, 7].

If they are to contribute meaningfully to health gains and realize their potential at scale, national CHW programmes require careful thought, planning and extensive support [8–10]. They need to be integrated into PHC systems whilst simultaneously embedded in and supported by communities [11]. The development and strengthening of national CHW programmes is made complex by the fact that they have prior histories and do not occur on a blank slate. Most countries have pre-existing community based initiatives of some kind, which more often than not exist on the margins of the formal health system. They are also neglected in health workforce planning, and deployed in fragmented, disease specific and uncoordinated ways [2].

How, then, should the strengthening of national CHW programmes be approached? McCord et al. [12] proposed that CHW programmes be regarded holistically as a sub-system of the overall health system, and using the World Health Organization (WHO) “building blocks” framework of a health system [13], they offer a comprehensive approach to CHW programme strengthening, encompassing the dimensions of service delivery, workforce planning, information systems, supply chains, financing and leadership and governance. A recent, wide ranging manual, developed for USAID’s Maternal and Child Health Integrated Program (MCHIP) provides similar guidance, whilst also emphasising the relational and process dimensions of national CHW programmes, such as planning, partnerships and scaling up [5].

Of the health system building blocks, *“arguably the most complex but critical”* [13] is that of leadership and governance, the building block which enables and holds the others together. Leadership and governance are not easy concepts to pin down. WHO defines them as “*the oversight and guidance of the whole system, public and private, to protect the public interest”,* and includes *“ensuring strategic policy frameworks exist and are combined with effective oversight, coalition-building, the provision of appropriate regulations and incentives, attention to system-design, and accountability”* [13]. In this definition, leadership and governance are focused on overall structures and design, generally at national level, with some attention paid to processes (such as coalition building).

A newer generation of approaches take a broader view of governance and leadership as not just a property of national governments, but as distributed within systems, involving an array of actors, and as straddling design and implementation. For example, Brinkerhoff & Bossert [14] bring into focus the roles of providers and citizens in governance relationships. From the field of implementation science, the PARIHS (Promoting Action on Research Implementation in Health Services) framework, foregrounds the leadership and governance of implementation [15]. Hill and Hupe’s multiple governance framework proposes three forms of governance, focused on overall design and setting of rules (constitutive governance), detailed decision making (directive governance) and managing implementation (operational governance) [16]. Drawing on similar concepts, Abimbola et al. [17] outline a multi-level governance framework for plural PHC systems that is centred on the decision-making of, and accountability relationships between, local providers and communities, situated within overall national frameworks.

Reflecting these currents of thinking, Lewin and Lehmann [18] approach the issue of CHW programme governance as establishing the architecture, relationships, decision making and participation structures of programmes. This includes whether CHWs should be part of the formal health system or managed separately, the extent of decentralised decision making, and mechanisms of community participation. They emphasize that *“because CHW programs are located between the formal health system and communities and involve a wide range of stakeholders at local, national, and international levels, their governance is complex and relational.”* [18] There is also an overlap of governance and management, where the latter is *“more concerned with running or implementing programs”* [18].

Together these various ideas on CHW programme governance and related concepts such as leadership, strategic management and implementation, point to a set of distributed functions that span policy development and systems design, structures and mechanisms for coordination and participation, and programme implementation. They are not just concerned with the “what” of CHW programme policy, but also with the “how” of implementation and scaling up. Specifically, CHW programmes require engaging a more complex and plural set of players — extending into communities — than is normally the case with other sub-systems of the health sector.

On the whole, however, thinking on CHW programme leadership and governance is undeveloped. Where it exists it is oriented to questions of national policy and design of programmes, and much less on the sub-national dynamics of decision-making, policy adaptation, refinement and implementation within health systems. In many health systems, managers and implementers are having to implement reforms to community based health systems. Faced with new, often incompletely elaborated national mandates, how do they turn CHW programme policy into reality?

Drawing on notions of CHW programme leadership and governance as distributed in health systems and as more than the constitutive (design) dimensions [16], this paper provides an empirical case study of reforms to the community based health sector in South Africa. It asks the question: What do provincial experiences with the adoption and implementation of the Ward Based Outreach Team (WBOT) Strategy in South Africa offer for an understanding of the governance and leadership of CHW programmes at scale? Based on case studies of the early implementation of the community based strategy in three provinces (North West, Western Cape and Gauteng), an inductive cross case analysis was conducted with the objective of identifying leadership and governance roles and tasks required in national CHW programmes.

## Background

South Africa is a middle income country, providing health care through a tax funded public health system to 84% of the population, with the remainder receiving care in a parallel private sector, funded by costly private health insurance, and entrenching massive inequities in expenditure on health. However, access to nurse-based PHC is reasonably good, with 90% of South Africans living within 7 km of the nearest public clinic [19]. Despite this, South Africa still has very high levels of avoidable mortality caused by burdens of both communicable and non-communicable disease, as well as injury and violence. Much of this burden is preventable, and there is an urgent need to strengthen the preventive and promotive responses of the PHC system.

To this end, as part of a broader set of reforms, South Africa is seeking to reorient a loosely structured and highly diverse community care system that emerged organically around HIV (human immunodeficiency virus) and tuberculosis (TB), into a formalized, comprehensive and integrated CHW programme. The community care system was for the most part implemented through community based organisational intermediaries, many of whom were subsidised by government through HIV/TB budget lines. Inspired by the success of the Brazilian Family Health Programme, a “PHC Re-engineering” Task Team was appointed by the Minister of Health in 2010 to develop proposals for the reorganisation of community based services. In a “Discussion Document” [20] the Task Team outlined a set of proposals for the establishment of “Ward Based Outreach Teams” of CHWs, led by professional nurses (referred to as “Outreach Team Leader”), linked closely with other community based providers (e.g. environmental health officers), and local PHC facilities. They would be assigned to electoral wards, responsible for a defined number of households and accountable to the local health facility. The Discussion Document also proposed that CHWs be incorporated into the health system as part of the formal health workforce. The roles of teams were to be comprehensive: extending beyond HIV/TB to include maternal-child health and chronic non-communicable diseases; with preventive and promotive, in addition to care orientations, and mobilising cross-sectoral collaboration on the social determinants of health.

South Africa has a quasi-federal political system where the national sphere sets policy and nine provincial governments (and their elected legislatures) bear the main responsibility for the delivery of health services. It is thus a system with considerable decentralised authority and decision making. With respect to the Ward Based Outreach Teams (WBOTs), the national Department of Health (NDOH) defined an overall model and roles, developed a curriculum (with the ultimate goal of national certification), provided initial training and designed a routine monitoring system linked to the national District Health Information System. It stopped short of providing ring fenced funding (as it had with other national priority initiatives), and the detailed design and implementation of the WBOTs strategy was left to provinces, which proceeded to adopt and adapt the strategy in varying ways and at different paces. While a formal WBOT policy is still in the process of being finalised, the concept is firmly anchored in the White Paper on National Health Insurance (NHI) and the subject of system strengthening initiatives in “pilot” NHI districts across all nine provinces [21].

## Methods

### Primary case studies

The case study method is an investigation of a real-life and contemporary phenomenon with reference to its context [22]. The primary case studies were conducted over a 1 year period in 2012/13 in the North West, Western Cape and Gauteng Provinces. The three case studies formed part of a national researcher collaboration, funded through a number of sources, to describe the “what” and “how” of early implementation of the WBOT strategy at provincial level. The North West Province was selected because of its role as a “revelatory” case [22] of successful early implementation. The two other case studies (Western Cape and Gauteng) were embedded within existing relationships and projects of the researchers in these provinces, and selected because of this. The scope and intensity of data collection was thus different in each province (Table 1). In the Western Cape, additional funding from the provincial government allowed for a fuller appraisal. In Gauteng, on the other hand, a combination of fragmented implementation and limited resources for the study resulted in the focus on one district only, and was the smallest of the three case studies.

**Table 1:** Provincial contexts and data collection

Province	North West	Western Cape	Gauteng
Population	3.7 million mostly rural	6.2 million mixed urban/rural	13 million mostly urban
Per capita GDP (US$ 2010)^a^	6700	8700	9700
Districts/Sub-districts	4 districts 19 sub-districts	6 districts 32 sub-districts	5 districts 27 sub-districts
Sampling	Provincial level plus all 4 districts	Provincial level plus 2 districts in depth	One pilot district
1. Interviews			
*Senior Management*			
Individual interviews	4	23	1
Group interviews	1		
*Middle and frontline management*			
Individual interviews	14	26	4
Group interviews	1		
*WBOTs/NGOs*			
Individual interviews	9	35	1
Group Interviews	7	5	1
*Community members*			
Individual interviews		16	
Group interviews		5	
*Other* ^b^		13	
Total	27 individual, 9 group interviews	113 individual, 10 group interviews	6 individual, 1 group interview
2. Observations	Provincial Task Team	23 CHW home visits	Health Post structure
3. Document reviews	Policies, plans, project reports, parliamentary speeches, training guides		
4. Routine and audit data	Household profiling; audit of CHW workers	Database of CHWs and NGOs	

^a^Source: https://en.wikipedia.org/wiki/List_of_South_African_provinces_by_gross_domestic_product_per_capita

^b^Other = other sectors (education, social development), traditional healers, private providers

Despite these differences, each case study was concerned with documenting the same phenomenon, and drew on jointly developed tools and methods, adapted to local needs and resources. The case studies mapped system components (based on a health system framework [23]) relevant to the new policy, and assessed implementation contexts and processes [24]. Data collection included in-depth interviews (audio recorded, transcribed and analysed thematically) with a cross section of health system actors, from decision-makers to front-line, observations of practices and processes, patient and community interviews, reviews of documentary sources and analysis of routine data (Table 1).

The North West and Western Cape case studies involved teams of researchers from diverse backgrounds, who analysed data in an iterative process, starting with individual data sources, followed by triangulation and convergence towards key themes. Being more limited in scope, the Gauteng study was analysed by two researchers. All three case studies undertook careful processes of member checking (feedback and discussion with respondents) before finalization. The trustworthiness of the findings was enhanced by the collective experience and tacit knowledge of the research teams, able to contextualize and make sense of findings. Full accounts of the provincial contexts, case study research strategies and findings are reported elsewhere and summarized below [25–27]. Each case study received ethics clearance from an institutional review board.

### Overview of cases

#### Case study 1: North West Province

The North West Province was an early and enthusiastic adopter of the WBOTs Strategy. Within a year of the national proposals, the Province had begun implementation and by the time the case study was conducted (late 2012), pilot teams were established in all sub districts and more than 40,000 households had been visited. Scale up has continued since, and by 2015 [28], more than 300 WBOTs were active across the Province, giving it the highest coverage of wards (72.6%) in the country. The case study sought to identify the factors underlying the successful and rapid implementation of the strategy in the province. The key insights offered were the active provincial strategies of implementation adopted and the forging of common collective visions, against a backdrop of well-established district and sub-district structures.

#### Case study 2: Western Cape Province

One of the authors (HS) formed part of a team commissioned in 2013 to conduct a situation appraisal of the existing NGO-contracted home and community based care services in the province, as part of a broader provincial strategic planning process (referred to as Healthcare 2030). Up to then the province had resisted the national WBOTs proposals, specifically opposing moves to do away with NGO intermediaries and incorporate CHWs into the provincial staff establishment. However, the Healthcare 2030 Strategy ultimately proposed far reaching changes to the community based health services in line with the national strategy [27]. The situation appraisal thus identified the key design challenges to reshaping the existing community based services to the new goals in a setting where political and stakeholder commitment to the new ideas was mixed.

#### Case study 3: Gauteng Province

In contrast to the other two provinces, the Gauteng provincial authorities did not take an active stance for or against the WBOTs policy, essentially acting as a conduit for the communications from the national department to the five districts. This province had an established infrastructure of district family medicine practitioners, linked to the three universities, who had already been experimenting with different models of community oriented PHC. The districts were asked to integrate the WBOT strategy into their existing models and by 2015, 55% of wards had WBOTs [28]. One of the co-authors (NN), conducted an assessment of this integration and assimilation process in one district, Sedibeng, selected as an initial pilot site for implementation of the WBOT Strategy. This case study provided insight into how district actors who have already reorganised their community-based services engage with top-down mandates, and the role of local stewards in negotiating the fit between the two.

### Cross case (secondary) analysis

A qualitative, descriptive, cross case analysis of leadership and governance roles was conducted after the three case studies had been completed and written up. The cross case analysis was an embedded unit of analysis in that it focused specifically on the issues and challenges faced, and the strategies adopted, by provincial and district policy makers and managers as they engaged with the new national mandate (the “case”). Drawing on the opportunity offered by three distinct sets of experiences, attitudes and contexts, the analysis was able to ensure maximum variability in what Yin [22] refers to as the “replication logic” of sampling — the study of the same phenomenon in different contexts. In an inductive process, each case study report (including findings, discussion and conclusion) was read and specifically coded for potential leadership and governance roles/tasks/challenges/strategies. Informed by policy analysis approaches [29], codes were then categorised into broad themes (e.g. provincial adoption of policy, actor roles and responsibilities). In this manner, key findings from each case were surfaced and patterns matched with those of the other cases. Each case added unique insights as well as confirmation of patterns in the other cases. From this, a set of governance or leadership tasks or roles for CHW programmes at scale were formulated.

The cross case analysis was conducted by the first author (HS), who had led two of the original case studies, while the co-author (NN), who had led the third case study, provided a critical mirror on the plausibility of the analysis. The analysis remained at a descriptive level, and did not seek to build theory on cause-effect relationships (e.g. what explains success or failure of implementation and/or governance and leadership?). It also did not formally test rival formulations of roles, but drew substantively on the findings and interpretations of the individual case studies, which themselves had undergone extensive validity checks.

## Results

The key issues facing, stances adopted and strategies deployed by the provincial and district policy makers/managers arising from the three case studies are summarised in Table 2. They have been grouped into the broad themes of provincial policy adoption and formulation; reallocation of roles and responsibilities; the development of new systems; and leading and managing change. These are described in more detail in the narrative that follows.

**Table 2:** Key leadership and governance themes identified in case studies of WBOTs implementation

Broad L&G function	Province		
	North West	Western Cape	Gauteng
Policy formulation/adoption	Long standing and widespread support for the district health system and PHC led to ready acceptance and early adoption of the policy	A well-established and reasonably governed system of NGO contracting for community based care perceived as different to national WBOTs strategy and led to minimal initial adoption, but later formulation of a comprehensive strategy	District based nodes of innovation, led by family physicians and following unique local designs (“health posts”), led to a complex negotiated process of accommodation and adaptation of the WBOTs policy at local level
Reallocation of roles and responsibilities	In all three provinces the reorientation of community based services implied new roles, relationships and mindsets amongst all role players in the community based, PHC and district health systems - local health facilities and managers had to play new oversight and coordination roles and be willing to allocate resources (staff, space) in support of teams - new relationships had to be developed with communities and community structures - the roles of the NGO sector had to be redefined - (sub)-district systems had to play a stronger priority setting, planning and monitoring role		
Development of new systems	In all three provinces, community based services have existed on the margins of the health system, with poorly developed and integrated human resource, financing and information systems. Greater expectations of performance of the community based sector have demanded changes in these systems: - payment of CHW stipends shifted from NGOs to government payroll systems to ensure regular payment (in two provinces) - improved support and supervision from professionals - new curricula and training processes instituted for standardised and comprehensive roles - new M&E systems developed that are aligned with new roles and integrated into the routine district health information systems, and piloting the use of mHealth However: - Financing is still largely from special budget sources (such as HIV/AIDS and TB conditional grants), received from national government, and only partially integrated into core provincial resource allocation mechanisms - Remuneration, conditions of service and career pathing for CHWs have not been adequately addressed - Recruitment and funding of Outreach Team Leaders a key factor in future sustainability		
Leading and managing change	Rapid adoption of the strategy followed a common collective vision about WBOTs that led to strong leadership of the process at district and sub-district levels. This was accompanied by deliberate scale up processes: planning, piloting, community “dialogues”, implementation support structures, including feedback and accountability	At the time of the case study, the province was still in policy formulation stage. In subsequent months there was an incremental process of negotiating new roles and modes of delivery with NGO partners, and developing new training and M&E systems. Piloting of comprehensive roles planned at district level (in the NHI pilot site).	Changes happened prior to new policy. The leadership role of family physicians in partnership with DHS management was key, and led to the development of a unique district model. Involved extensive local alliance building (including mobilising local financial resources for implementation)
	All provinces face the challenge of generating political, including budgetary commitment, and developing the case for greater investment and resources for WBOTs		

### Policy adoption and formulation at provincial level

Given the relatively loose, unfunded mandate from the national sphere, provincial attitudes to implementation of the WBOTs strategy differed. Provincial leaders in the North West Province (NWP), where political and senior managerial commitment was high, regarded it as an affirmation of long standing values and orientations towards PHC in the province. As pointed out by one manager: *“The elements of PHC re-engineering have long been implemented in the North West… The official adoption by the national department of the PHC re-engineering as a model upon which to push our service delivery ….confirms that what we have been doing is correct, and therefore strengthens what we were doing…”* (District Manager, NWP). Similar understanding and ownership were evident across all levels of the system, including amongst the CHWs themselves. The fit of the new policy with existing values and approaches was thus unproblematic in this province.

In the Western Cape (WC), the WBOTs strategy was seen as distracting from unfolding trajectories and “ways of doing things” in community based services, and had mixed support in the province. Senior managers initially rejected the national PHC Re-engineering proposals, a stance regularly taken by this province in relation to the national sphere. However, the situation appraisal documented widespread support at district and sub-district levels for a re-organisation of the community-based sector towards more comprehensive and population oriented approaches. In line with a wider provincial commitment towards “wellness” and “wellbeing”, the province proposed an extensive re-organisation of its community-based services in the Healthcare 2030 strategy. However, it retained the service delivery model of non-governmental organization (NGO) intermediaries: *“The NGO model has a lot to offer, let’s figure out how to do it better.”* (Senior Provincial Manager, WC). Since then, it has focused on negotiating an incremental widening of CHW roles with the NGO sector, and is piloting new approaches to delivery in various parts of the province, including the nationally supported NHI pilot site.

In the Sedibeng District of Gauteng Province (GP), the adoption of WBOTs confronted an already developed local model of outreach called “health posts”, led by a Cuban-trained family practitioner. Health posts are basic physical structures, often constructed with resources mobilised from local communities, as satellite delivery sites for clinics and community health centres. The health posts are staffed by a professional nurse (recruited from a pool of retired nurses) and a team of CHWs, and bring preventive services and chronic disease follow-up and distribution of medicines closer to the community. When the WBOTs were introduced *“there were meetings and we were informed about what national wants … we had already been having PHC Re-engineering, although we were calling it health posts, but they said the name must change, it must be PHC Re-engineering, then that’s it”* (Sub-District Manager, GP) *“The whole project had to be re-adjusted according to what the [national] minister wanted.”* (District Manager). The district did not want to do away with the health posts because *“communities are already comfortable with that system [health post]. If we now start to close or change they might feel we are really playing games with them”* (District Manager) and settled on a hybrid model where health posts became referred to as “Ward Based PHC team sites”.

### Reallocation of roles and responsibilities

The community-based health sector in South Africa developed from the late 1990’s as a government supported, NGO-based service focused on provision of care and support for people with HIV and TB. With varying degrees of formality, it related to a diffuse set of players including hospitals, step down and palliative care facilities, HIV/TB providers, welfare sector and other NGOs. It did not therefore emerge as a structured extension of the PHC system, and government funding for NGOs was channeled through HIV/TB programmes.

The WBOTs strategy proposed a shift towards comprehensive CHW roles and pro-active engagement with households and communities, with a primary link to the PHC system. These involve a significant reconfiguration of local relationships between PHC professionals, CHWs and communities. Health facilities and sub-district managers have to play new oversight and coordination roles and be willing to allocate resources (staff, space) in support of outreach teams; they need to engage more actively with the diverse array of actors within communities, and shift from mindsets of treatment to prevention and promotion. Prevailing organisational cultures are generally not in support of this.

In the North West Province, the expectation that PHC clinics would provide the WBOTs team leaders from within their own staff establishment was met with surprise and in some instances, resistance: *“I was not aware that he [the team leader] will be out of the facility permanently because I expected him to come back and to still allocate work to him”* (PHC facility manager, NWP) In both this province and the Western Cape the dominant attitudes of PHC professionals towards CHWs was to regard them as subordinate cadres and not as agents with independent knowledge of community life and capable of judgment and discretionary action. While the role of the team leader as a support system was viewed very positively by CHWs in the North West, relationships with health facility staff remained precarious and a source of considerable dissatisfaction. Team members were constantly under pressure to work in clinics: *“If there is a shortage of staff like this month … they were taking us to work that clinic and then many go to work in that clinic. That is what happens.”* (Outreach Team Leader, NWP).

The case of Sedibeng demonstrates how local leadership from the sub-district management team and the family medicine practitioner, attuned to community oriented PHC, can successfully mediate these new relationships. They also strengthened the hand of the outreach teams through the health posts, which provided an autonomous physical space for WBOTs that did not rely on the goodwill of PHC facility staff, whilst also indirectly addressing the need to alleviate the pressure from overcrowded PHC clinics. However, it introduced a new line of accountability (the professional nurse at the health post reports to the facility manager at the PHC clinic).

A more visible and systematic approach to households and communities requires a level of buy-in and participation that was not necessarily the case in the more limited care and referral system of the past. As explained in Sedibeng: *“Implementation of PHC re-engineering is a real community-based process. You have to talk to political leadership. You have to talk to officials in the municipality. You have to talk to other prominent figures. You know we even went to the ministers of different religions. So you really have to be as participative with the community as possible. If you don’t then you miss out completely”* (Senior District Official, GP). In the North West Province, “community dialogues”, involving a wide cross section of players, were a key part of the implementation process and established community participation and inter-sectoral action as valued elements of the strategy. *“The implementation dialogues must be carried out for the community to be aware of what is going to happen and they must accept because if they don’t that will cause us unnecessary challenges. ”* (Outreach Team Leader, NWP) Similarly, in the Western Cape, community members interviewed welcomed a re-organisation of roles but emphasized the need for greater participation. *“Communities … can play a big role in if they were educated about the new vision and have knowledge about the new system.”* (Community member, WC). None of the three provinces had considered formal community oversight roles, such as through clinic committees, of outreach teams.

Despite emerging from an NGO-driven system, the WBOT strategy is silent on the role of NGOs, and several provinces have opted to do away with NGO intermediaries and contract directly with individual CHWs. While some NGOs may disappear others will continue to have a community presence and will form part of the array of local actors to be engaged in community health systems. Where NGOs remain as contracted agents deploying CHWs, such as in the Western Cape, their organisational relationships also have to be redefined. An NGO partnership system requires capacity for managing contractual relationships that includes not only financial accounting and performance monitoring but also the trust relationships necessary for effective cooperation in a plural environment. The Western Cape situation appraisal recommended that contracting of NGOs shift to sub-district authorities, away from the more remote and disconnected District Community Based Services division, as in the past. This will also allow for greater priority setting and planning at this level.

### Development of new systems

Following the publication of the PHC Re-engineering Discussion Document (which spelt out the core concept of the team approach and roles), the national Department of Health commissioned an inter-related set of processes that included the design of a national work-based training curriculum (through a national accrediting body), indicators and a routine reporting system through the District Health System, and the development of in-service training packages.

These elements formed the leading edge of reorganised community based services in the provinces and their alignment facilitated implementation, where this was observed in North West and Sedibeng. However, several key human resource and related financing questions remained unresolved at national level, and were thus implicitly delegated to provincial players. These included the employment status and remuneration of CHWs, the roles of NGOs, and the mobilisation and funding of nursing personnel as team leaders.

In a process emulating other provinces (begun in KwaZulu-Natal, a province not studied), both Gauteng and North West decided to move away from payment of monthly CHW stipends through NGOs, experienced as unreliable and frequently interrupted, to direct payments through the government payroll. As indicated, the Western Cape chose to remain with the NGO contracting system that functioned relatively well in this province. However, without the additional funding nationally, the levels of stipends were not increased and remained well below the entry level wage in the civil service. The Western Cape case study documented a very high turnover of CHWs, especially in the urban areas as a result [29], and retention and stability of WBOTs remains a key issue.

In the North West, which has scaled up WBOTs despite the absence of additional funding, the strategy was integrated into existing district and sub-district resource allocation, planning and monitoring mechanisms. As a senior provincial manager indicated, “*districts were being encouraged to “work differently” within the PHC re-engineering framework and obtain necessary budget accordingly.”* This was accepted at lower levels: *“If it’s part of our mandate, then it’s in the equitable share [core budget]. It’s a good thing because we will own it 100% and we’ll plan and implement it accordingly.”* (PHC Facility Supervisor) In Gauteng, the provincial government provided budgets to Districts to recruit retired nurses to support teams. However, the health post component continued to rely on local resource mobilization: *“We also had to ask for donations, because it was a mandate but it was an unfunded mandate. So they said we should ask for donations from business people or from wherever.*” (Sub-district manager, GP).

The design of integrated health system support systems is perhaps the best recognised of the leadership and governance roles in CHW programmes. However, while national policy processes provided the overall design and core idea of the WBOTs, these processes remained incomplete and had to undergo further development with implementation.

### Leading and managing change

The North West Province provided the clearest example of the sub-national leadership required to catalyse changes to community-based services systematically and at scale. The primary case study [27] identified these as an inter-related set of processes that included:

- The forging of a collective vision for the new strategy that built on prior history and values and that led to distributed leadership and ownership of the new policy;
- An implementation strategy that ensured alignment of systems (information, human resources) and appropriate sequencing of activities (planning, training, piloting, household campaigns);
- The privileging of ‘ community dialogues’ and local manager participation in the early phases;
- The establishment of special implementation structures: a PHC Task Team (chaired by a senior provincial manager) to enable feedback and ensure accountability, and an NGO partnership that provided flexible support for implementation.

In the North West, a rural province relatively shielded from the dominance of tertiary care centres and medical schools, the values of PHC (such as community participation and inter-sectoral action) have found ready acceptance. In the Western Cape, community based services are still regarded by providers and frontline managers as a clinical extension of care in clinics and hospitals. Those seeking to implement the values espoused in Healthcare 2030 thus face the challenge of both building political commitment and achieving consensus on a different orientation. In contrast to the North West where a collective vision and support was evident and an important driver of change, views on reforms to community-based services in the Western Cape were more fragmented. As one interviewee said, *“The problem is that it is such a wide concept and each person interprets the concept in their own way … [they are] all on different pages. [I] don ’t think management understands it or is fully in agreement on what it should be.”* (District Manager, WC). In Sedibeng (and in Gauteng more generally), the leadership role of family medicine specialists, linked to universities, has played a major role in legitimating new forms of community oriented PHC. However, these initiatives have tended to remain local and therefore uneven across the province.

All three provinces face the problem of national political ambiguity towards the WBOT Strategy. The strategy features in all the key overarching reform statements (notably NHI), but is not backed by funding or developed yet as a specific policy. Despite the presence of routine information systems, monitoring and evaluation of WBOT implementation remains weak, and the demand for evidence is low.

A key problem is that implementing the WBOTs will require significant new investments, especially in the regularization of the employment of CHWs, but also in better support systems. In a middle-income country with a relatively well-developed and accessible facility-based PHC infrastructure, the added value of WBOTs will be in the preventive and promotive roles they can play. Opening up the fiscal space for this requires compelling evidence on the capacity of comprehensively oriented WBOTs to address disease burdens and the social determinants of health. Unfortunately, the evidence based from elsewhere, notably around the role of CHWs in child survival has limited applicability in South Africa. In the face of this, the focus has remained on disease specific community initiatives (notably HIV/TB), and on strategies to strengthen facility based services [30].

### Key leadership and governance roles

In all three provinces the adoption of WBOTs strategy involved an active process of making sense of, adapting and negotiating the fit with existing provincial realities. Provincial stewards were also faced with reconfiguring relationships within PHC and the district health system, and developing new management systems. Further, if the strategy is to be sustained at scale, they have to make the case for greater investment, build an evidence base, forge partnerships and alliances, and design coherent implementation strategies.

From the cross case analysis, four key leadership and governance roles for sub-national stewards seeking to strengthen CHW programmes and community based services have been formulated:

1. Negotiating a fit between national mandates and provincial histories and strategies of community based services;
2. Defining new organisational and accountability relationships between CHWs, local health services, communities and NGOs;
3. Revising and developing new, aligned and integrated planning, human resource, financing and information systems;
4. Leading change by building new collective visions, mobilising political, including budgetary, commitment and designing implementation strategies.

These roles include not just the design of new systems — the “hardware” of governance, but also managing actor relationships and generating political support — the “software” of governance [31].

## Discussion

Leadership and governance, the *“oversight and guidance of the whole system to protect public interest”* [13] is a relatively poorly researched and understood role in health systems. This paper provides one perspective on this phenomenon, through the lens of sub-national health system stewards seeking to strengthen community-based services in South Africa. Secondary analysis of three provincial case studies of WBOT implementation, representing different contexts, attitudes and moments in the policy process, provided the opportunity for understanding the governance and leadership of CHW programmes at scale. The findings have relevance for other health systems, especially those in the process of restructuring existing community based delivery systems that emerged from responses to HIV/TB [30]. With their complex stakeholder relations, CHW programmes provide a window into the dynamics of leadership and governance in health systems more generally. The paper also speaks to the role of leadership and governance in implementation [15].

By focusing on provincial and district actors and processes the analysis, firstly, confirmed Hill and Hupe’s contention [16], of the distributed nature of the leadership and governance function. Policy development and design of programmes are not a once off national process following a pre-determined check-list, but a dynamic, negotiated and iterative process involving actors at all levels. National mandates are just the starting point, and may be incomplete or even contradictory. If they are to be implemented, these mandates need to find their fit, through negotiation and adaptation, in the messy and crowded everyday reality of health systems [32]. Strong sub-national governance, able to adapt national frameworks to local conditions, set priorities and coordinate and mobilise local actors is thus key to ensuring sustained implementation of CHW programmes [33, 34]. Such processes inevitably result in distinct sub-national programme realities where even fundamental orientations may be shaped and reshaped at the local level (e.g. whether CHWs are to be viewed as a technical agent or community mobiliser). This requires recognising the essentially emergent nature of CHW programmes [35] and the appropriate role of national (and international) support in the face of this [7].

Secondly, with respect to CHW programmes, attention needs to be paid to the micro-level reconfiguration of roles, responsibilities and accountabilities — between communities, CHWs, PHC professionals and sub-district management — and how these affect the distribution of decision making and power, and therefore, prospects for equity [36]. In particular, the analysis revealed the complex relationship between community and facility based players and the importance of mechanisms that ensure that community based teams have a degree of independence and autonomy from facilities. Two well known CHW initiatives, the Mitanin Programme in Chhattisgarh State, India [37] and the Health Surveillance Assistants Programme in Malawi [32] manage and deploy CHWs through divisions of the health system that are separate but coordinated with the rest of the PHC system. The creation of health posts in Sedibeng and NGO contracting mechanisms in the Western Cape are also ways of structuring autonomy.

Whatever the mechanisms, restructured relationships require greater vertical integration and accountability of community based services through the formal health system. Equally important is strengthening the less formal and horizontal mechanisms of coordination and accountability within community health systems. Being able to build norms of responsiveness and answerability between local players in the wider community health system, despite the absence of formal lines of accountability, is a key element of local CHW programme leadership and governance. It requires the capacity to shift from modes of command-and-control (managing up and down) that are the dominant cultures within frontline service provision towards new relationships across organizational boundaries based on networking, cooperation and reciprocity (managing out) [27].

Thirdly, the analysis highlighted the strategic management role — defined as the capacity to look outwards, inwards and ahead simultaneously [38] — of steering change at scale through complex health systems. This involves deliberate and participatory change management processes, in which collectively held values and visions play an important part. It requires mobilizing political support, but also the management of a range of vertical and horizontal organisational relationships, [39] and an ability to learn-by-doing [38].

A limitation of the analysis is that it did not include a consideration of national leadership and governance. This would bring into focus the formal processes of policy development, resource mobilization and decision-making — the “constitutive” and “directive” governance [16] roles — required at this level. The paper also rests on the assumption of government as the main funder and initiator of community-based services. In many settings this is not necessarily the case, where government is just one agency amongst many, and where the governance reality may be very different to that described above [17]. Although all guided by the same overall purposes and involving common actors, the case studies varied in size and scope and were, in two instances, selected because of ease of access and prior knowledge and relationships.

## Conclusion

This analysis has contributed to an empirical understanding of leadership and governance functions in strengthening CHW programmes at scale. It highlighted the multifaceted, negotiated and distributed nature of these functions, spanning analytic, managerial, technical and political roles. It is beyond the scope of this paper to spell out the implications of the analysis for assessing or strengthening the leadership and governance of national CHW programmes. However, it does suggest the need for multilevel frameworks that provide both direction and flexibility, allowing for emergence and negotiation; and which combine the “hardware” of systems development with the “software” of change.

## Abbreviations

CHW: Community health worker
GP: Gauteng Province
HIV: Human immunodeficiency virus
NGO: Non-governmental organization
NHI: National health insurance
NWP: North West Province
PHC: Primary health care
TB: Tuberculosis
WBOT: Ward based outreach team
WC: Western Cape Province
